# Transarticular Cartilage Retrograde Intramedullary Pinning Surgery for Humeral Transcondylar Fractures: An Effective Approach to Preventing Postoperative Elbow Joint Contracture

**DOI:** 10.7759/cureus.79715

**Published:** 2025-02-26

**Authors:** Hiroyuki Taguchi, Kozo Moriya

**Affiliations:** 1 Department of Rehabilitation Medicine, Matsuyama Red Cross Hospital, Matsuyama, JPN; 2 Department of Orthopaedic Surgery, Kagawa Inoshita Hospital, Kanonji, JPN

**Keywords:** elastic fixation, elbow joint contracture prevention, humeral transcondylar fractures, less invasive soft tissues, protect the ulnar nerve, strong fixation, surgical method, transarticular cartilage

## Abstract

As a treatment for humeral transcondylar fractures, conservative therapy is prone to false joints. Although various surgeries are available, plate fixation is highly invasive to soft tissues and prone to contracture of the elbow joint, and percutaneous pinning results in weak fixation and tends to damage the ulnar nerve. In this report of two cases, we describe a new transarticular cartilage retrograde intramedullary pinning surgical method that is less invasive to soft tissues, has an accurate reduction and strong fixation, and does not damage the ulnar nerve. This surgery was performed on two patients with humeral transcondylar fractures who presented to our hospital. Both patients achieved good results, with strong bone fusion and sufficient elbow range of motion for activities of daily life.

## Introduction

Humeral transcondylar fracture is an intra-articular fracture of the elbow joint that often develops in the elderly. Surgery for elbow fractures often results in postoperative range of motion (ROM) restriction, which interferes with activities of daily living (ADL) [1-3]. Conservative treatment for humeral transcondylar fractures is less likely to cause bone fusion and is more likely to become a false joint [1,2]. In this work, we devise and introduce a surgical method that minimizes the likelihood of postoperative ROM restriction and facilitates improved bone fusion. We report two cases of humeral transcondylar fractures treated with our new surgical method for humeral transcondylar fractures after receiving approval from the Institutional Ethics Committee of Kagawa Inoshita Hospital.

This article was previously posted on Research Square on May 2, 2024, on the Preprints server.

## Case presentation

Case one

**Figure 1:**
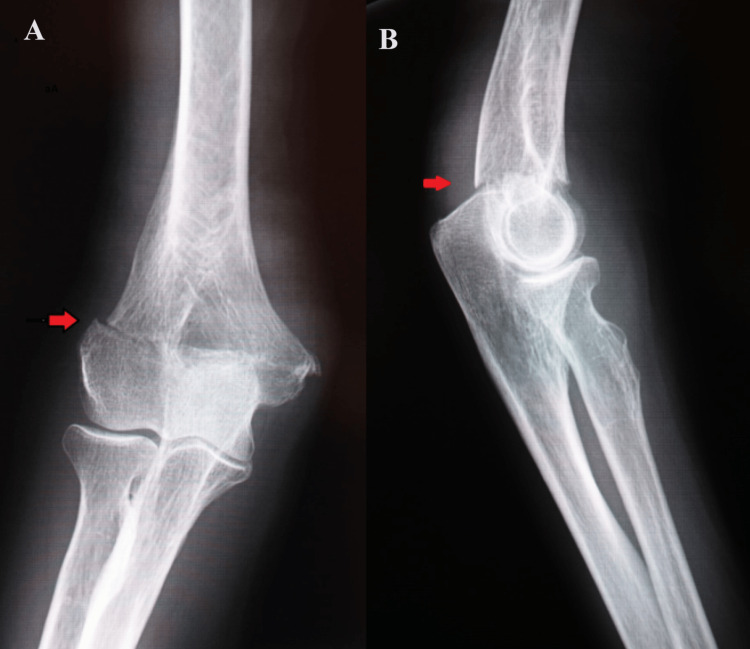
Right elbow preoperative X-ray images of case one (A) Anteroposterior view showing a transcondylar fracture of the right humerus; (B) side view showing an anteroposterior displacement of bone fragments in the transcondylar fracture of the right humerus.

An 81-year-old woman fell and injured her right hand, resulting in a transcondylar fracture of the right humerus (Figure 1). She was admitted to the hospital due to severe pneumonia and heart failure, during which she sustained injuries while in the hospital.

Transarticular cartilage retrograde intramedullary pinning surgery was performed six days after the injury. The following surgical methods were used: the right upper limb was placed on the trunk in the supine position, and the elbow joint was placed in the maximum flexion position. A 4-cm longitudinal incision was made on the dorsal side of the external condyle of the elbow joint. Two holes with diameters of 2 mm were drilled on the articular surface of the humeral external condyle. The tip of the Kirschner wire, with a diameter of 1.8 mm, was slightly bent to form an intramedullary nail, which was inserted through one of the holes drilled in the external condylar articular surface and advanced retrograde into the medullary cavity of the distal bone fragment. The medullary cavity of the proximal bone fragment was found at the fracture site, and the intramedullary nail was inserted into the proximal part of the humeral marrow cavity. The same operation was performed from the second hole.

**Figure 2:**
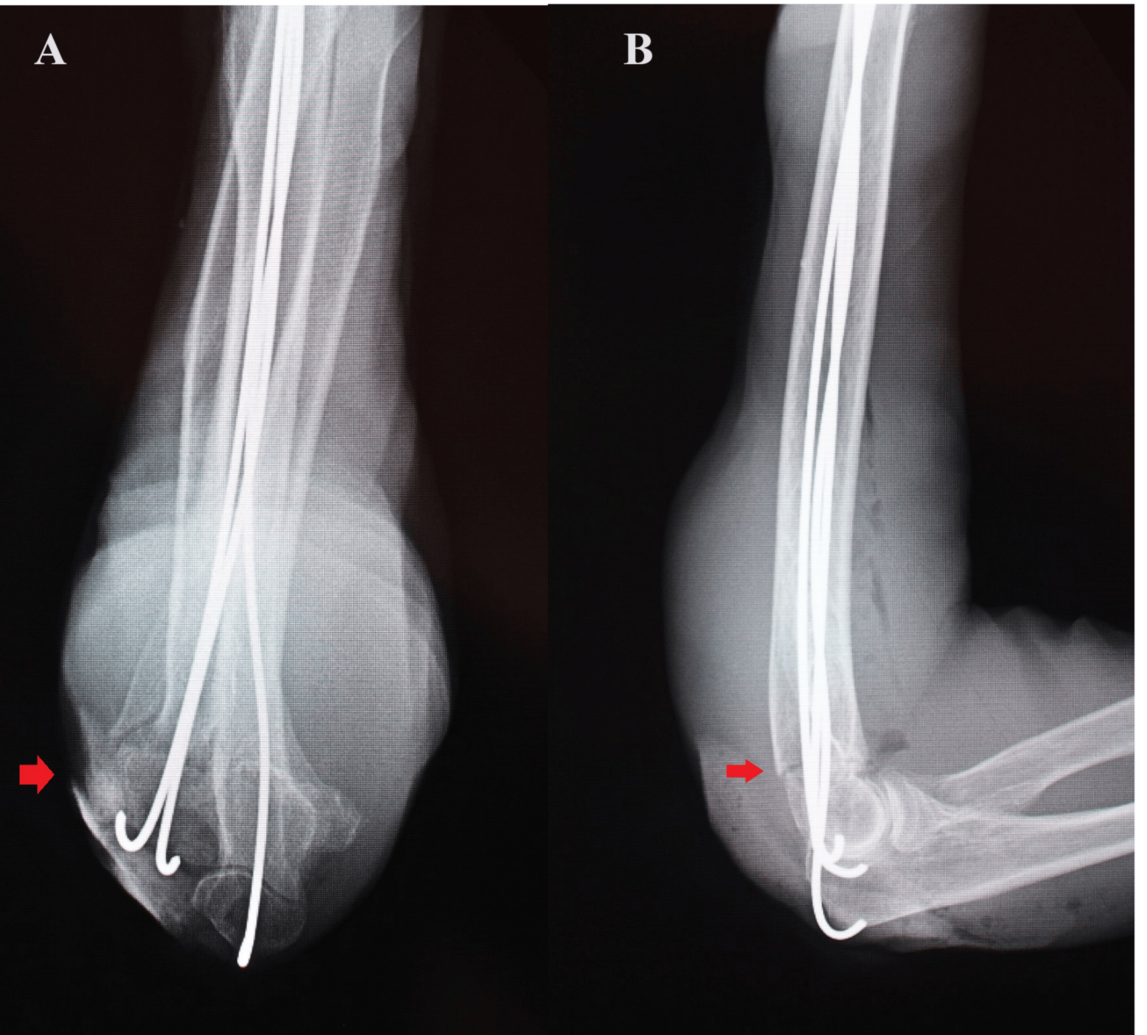
Right elbow postoperative X-ray images of case one (A) Anteroposterior view showing a precise reduction position of bone fragments in the transcondylar fracture of the right humerus; (B) side view showing a precise reduction position of anteroposterior displacement of bone fragments in the transcondylar fracture of the right humerus.

Subsequently, a 4-cm longitudinal incision was made on the inside of the olecranon. A hole with a diameter of 2 mm was drilled in the humeral internal condyle articular surface just inside the olecranon. We inserted one retrograde intramedullary nail on the inside in the same operation as on the outside. The fracture was precisely corrected with three intramedullary nails, and the elbow joint was firmly fixed, even when it was flexed and extended. No dislocation occurred (Figure 2).

**Figure 3:**
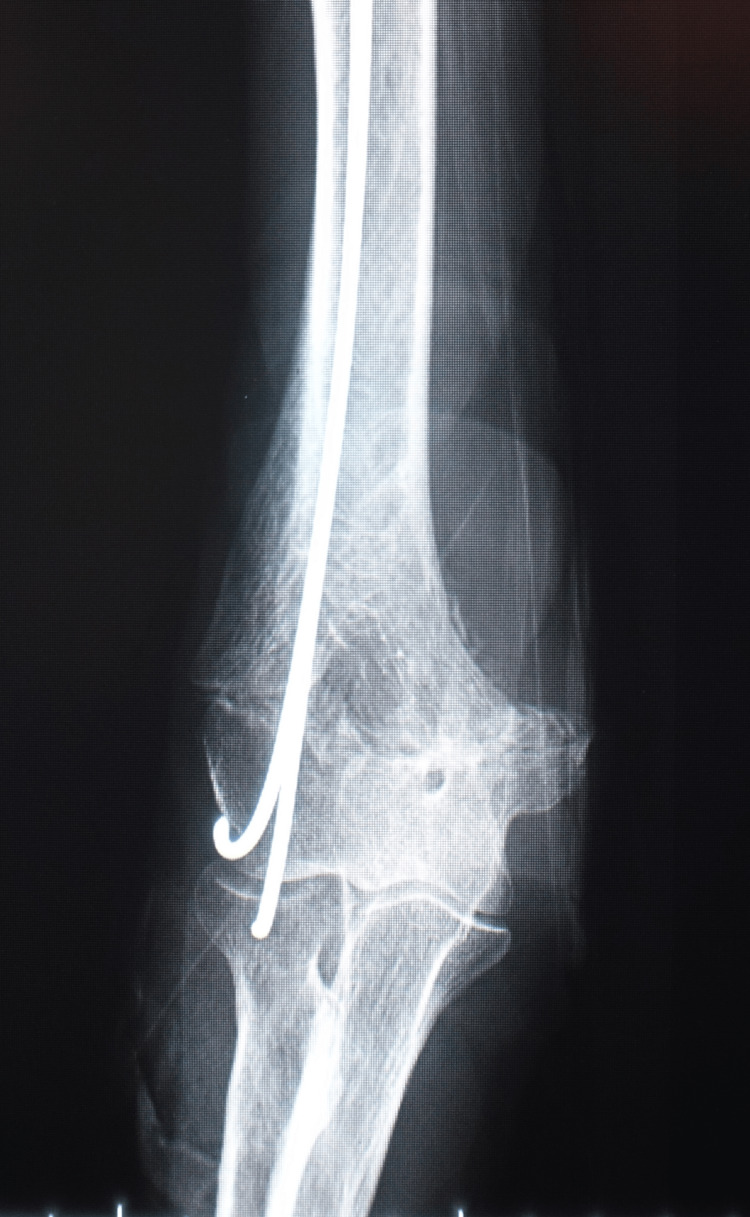
Right elbow X-ray images (anteroposterior view) of case one six weeks after surgery A small amount of false bone formation was observed, and the inner nail was removed.

The long upper limb cast was immobilized in a 110° flexion position and 90° external rotation for six weeks. The inner nail was removed six weeks after surgery, and automatic ROM training was started (Figure 3).

**Figure 4:**
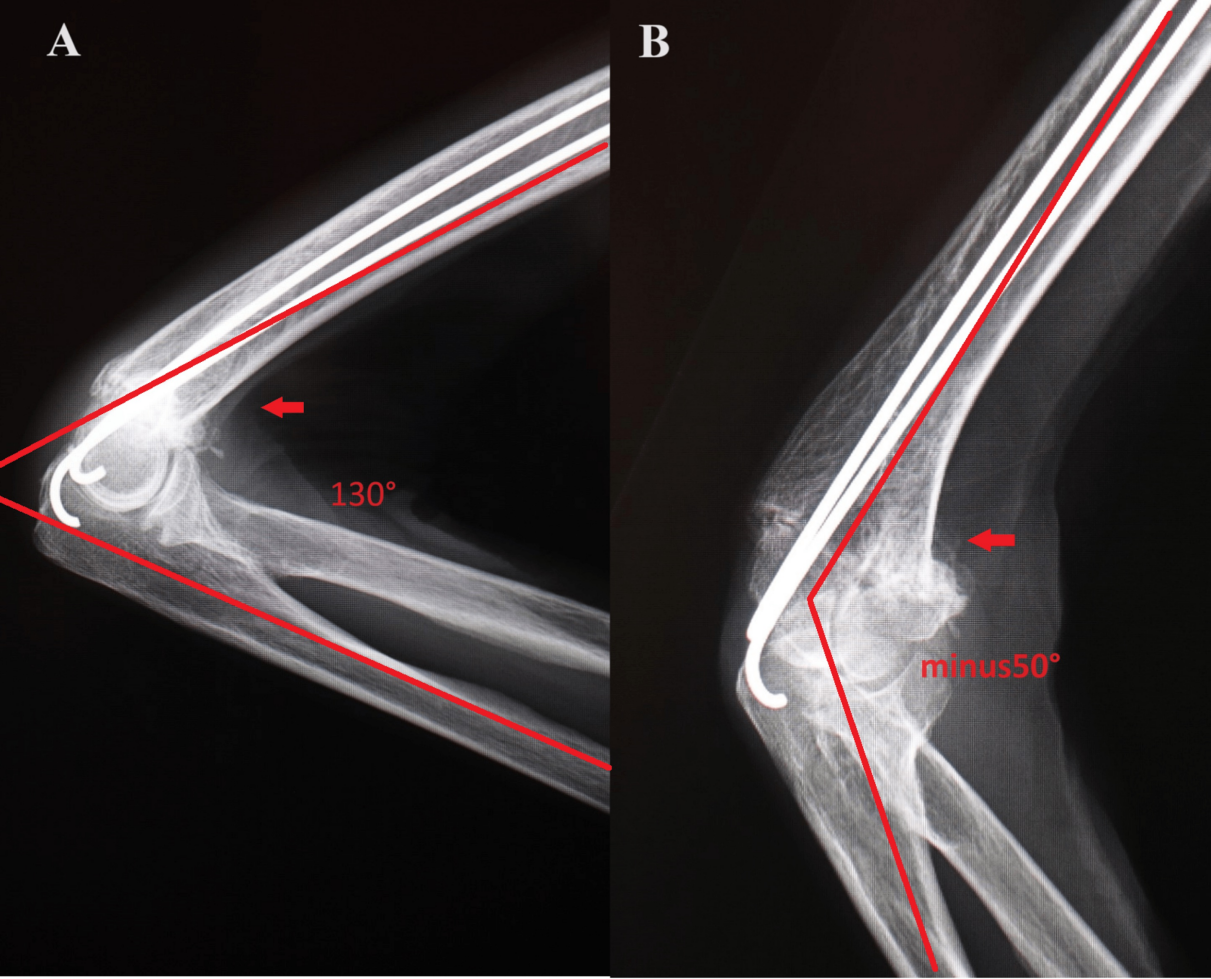
Right elbow X-ray images of case one three months after surgery (A) Side view showing range of motion of the elbow joint at 130° of flexion; (B) side view showing range of motion of the elbow joint at -50° of extension.

Three months after surgery, the elbow joint exhibited a ROM of 130° of flexion and -50° of extension (Figure 4).

**Figure 5:**
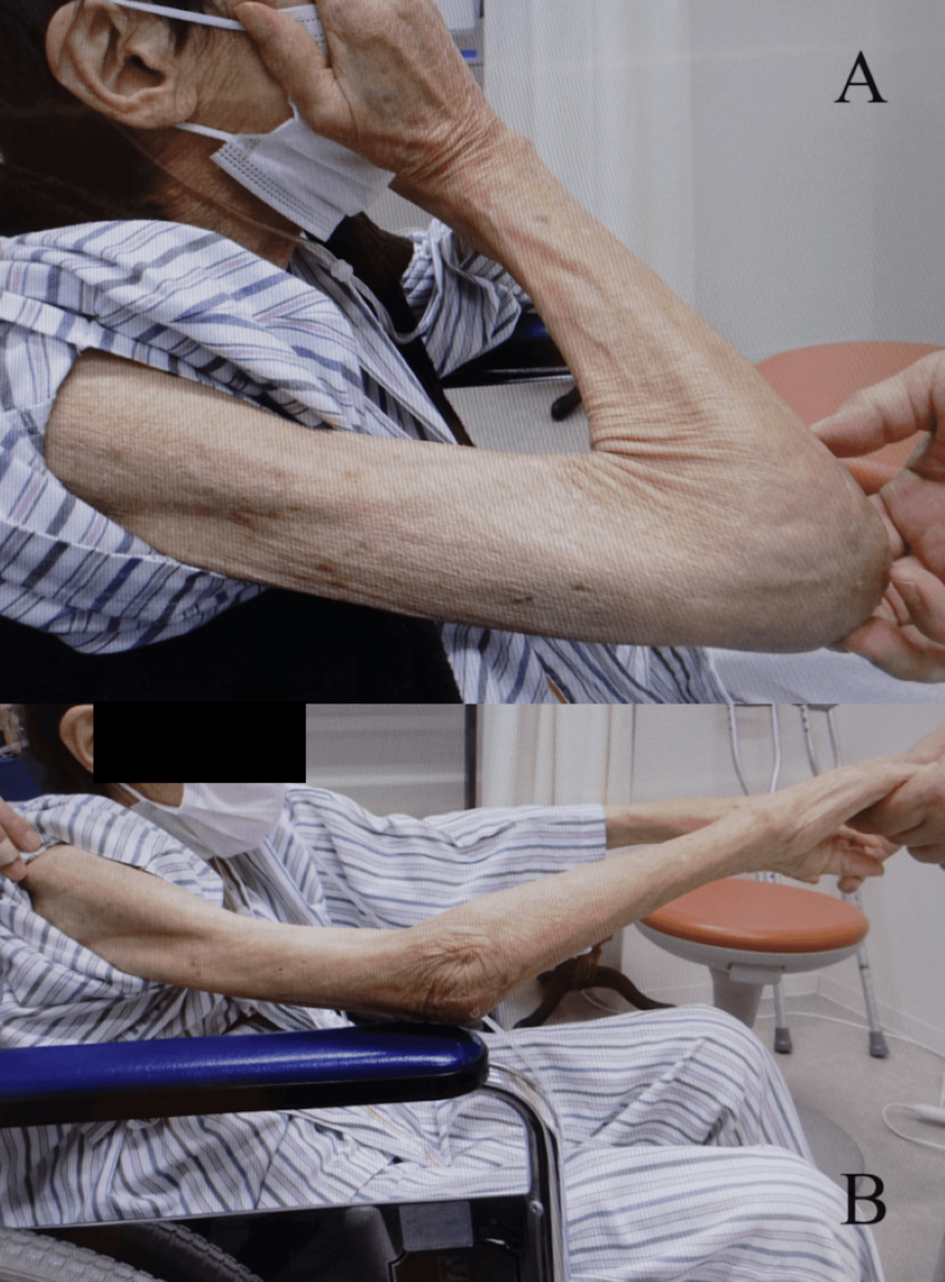
Case one at three months after surgery The patient was able to (A) wash her face and (B) extend her elbow joint.

The patient experienced no inconvenience in ADL, such as face washing (Figure 5).

Case two

**Figure 6:**
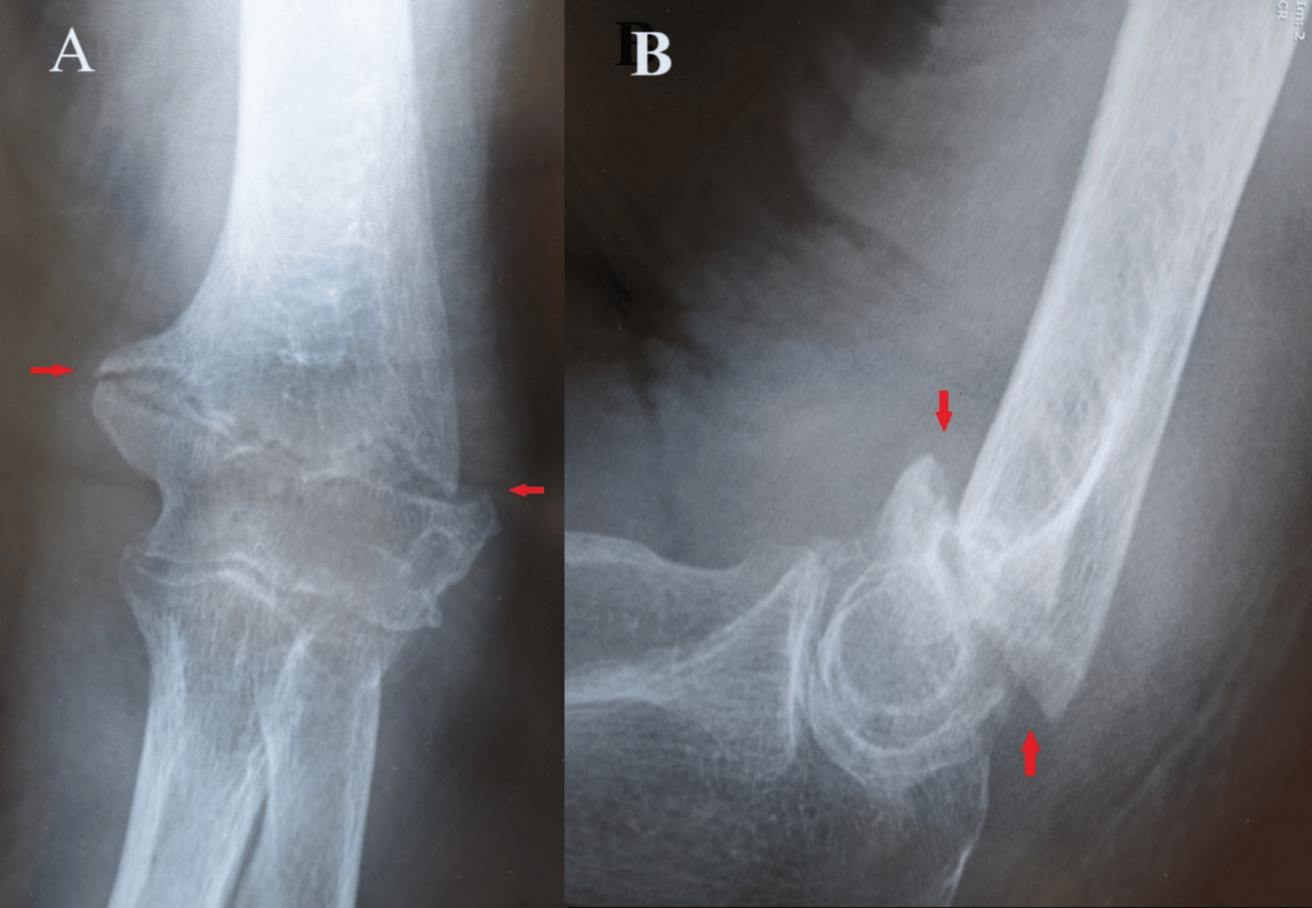
Left elbow preoperative X-ray images of case two (A) Anteroposterior view showing a transcondylar fracture of the left humerus; (B) side view showing an anteroposterior displacement of bone fragments in the transcondylar fracture of the left humerus.

An 80-year-old woman fell and injured her left elbow, resulting in a transcondylar fracture of the left humerus. She was wounded at home. There are no serious complications. She was placed in a cast for 12 weeks by a local doctor, but the bone did not fuse (Figure 6).

**Figure 7:**
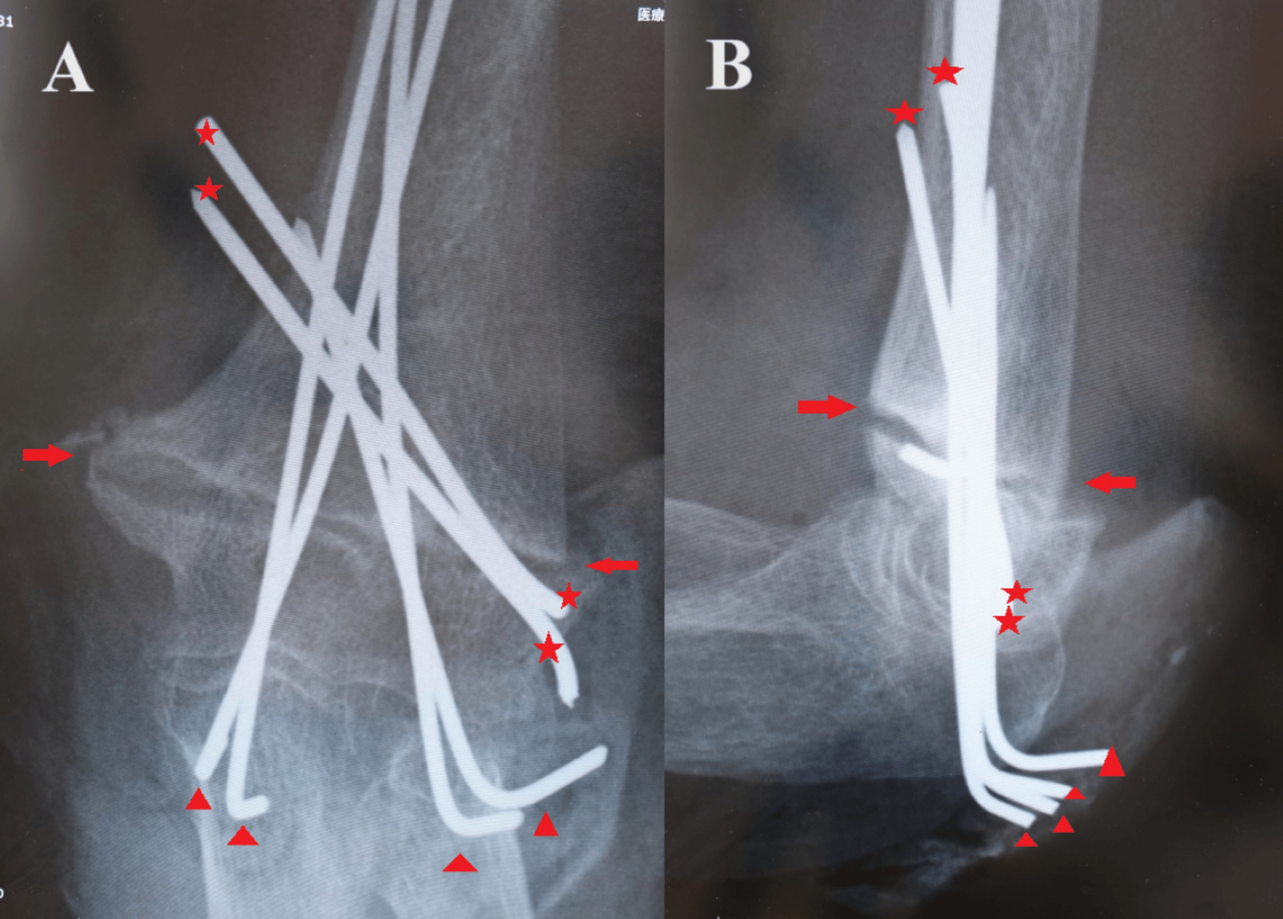
Left elbow postoperative X-ray images of case two (A) Anteroposterior view showing a precise reduction position of bone fragments in the transcondylar fracture of the left humerus (arrows); (B) side view showing a precise reduction position of anteroposterior displacement of bone fragments in the transcondylar fracture of the left humerus (arrows). Dislocation of the bone fragments was corrected percutaneously from the outside of the fracture line with oblique two wires (stars), followed by transarticular cartilage retrograde intramedullary pinning surgery (triangles).

She had suffered a humeral intercondylar fracture 12 weeks earlier and was undergoing conservative treatment. Transarticular cartilage retrograde intramedullary pinning surgery was performed three months after the injury. The following surgical methods were used: first, the dislocation of the bone fragments was corrected percutaneously from outside the fracture line with two 1.8-mm wires. Next, the left upper limb was placed on the trunk. The elbow joint was placed in the maximum flexion position, and longitudinal skin incisions were placed on the dorsal side of the external condyle and the dorsal side of the internal condyle. Transarticular cartilage retrograde intramedullary pinning surgery was performed with a total of four 1.8-mm Kirschner nails: two from the external condylar articular surface and two from the olecranon circumference, using the same methodology as in case one. The fracture was precisely corrected and firmly fixed with four intramedullary nails (Figure 7).

**Figure 8:**
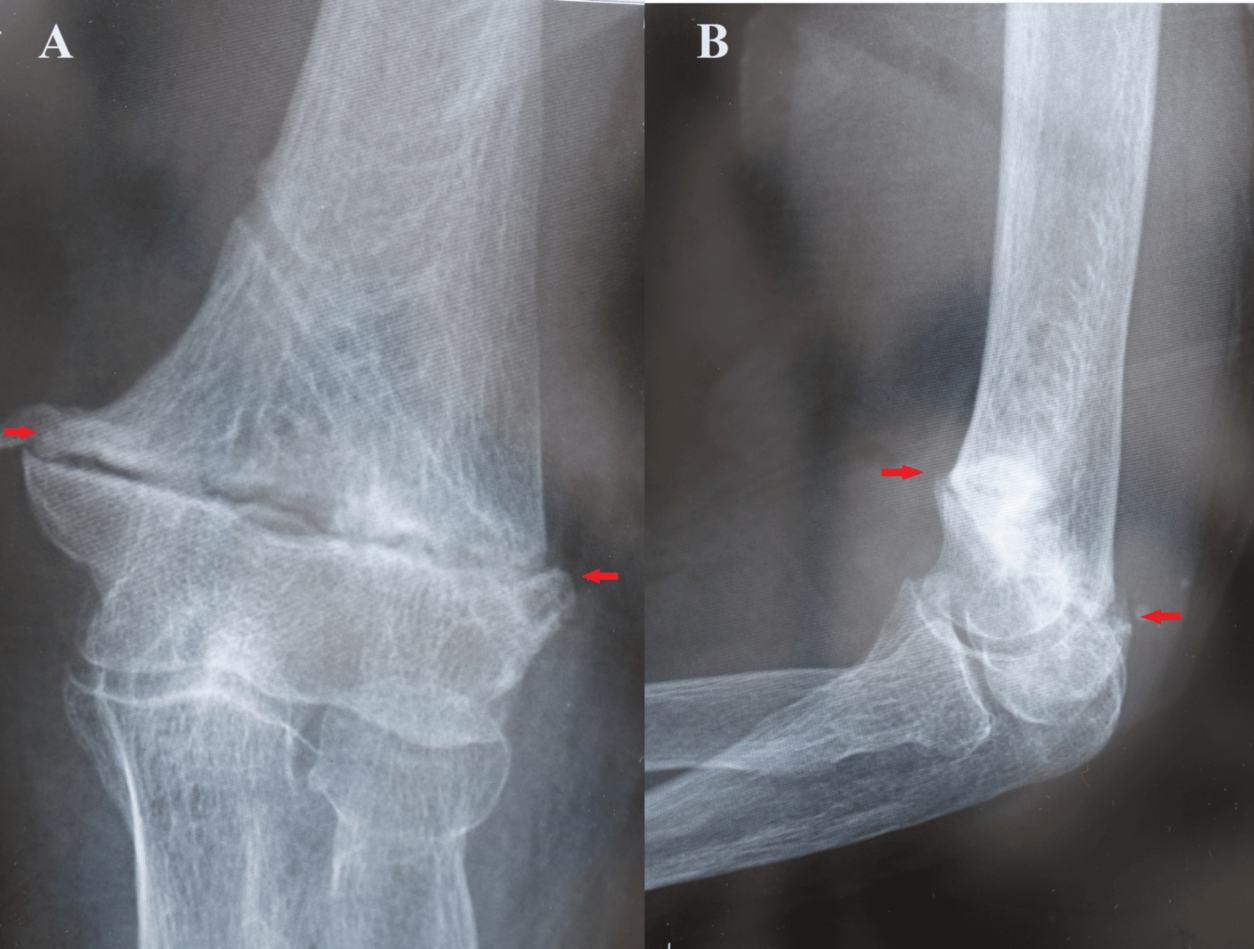
Left elbow X-ray images of case two at three months after surgery (A) Anteroposterior view and (B) side view showing that the fracture was firmly fused with good alignment.

The patient was placed in a long upper limb cast that was immobilized in a 110° flexion position and 90° external rotation for six weeks. Six weeks after surgery, all the nails were removed, and automatic ROM training was started. Three months after surgery, the fracture was firmly fused with good alignment (Figure 8), and the ROM of the elbow joint was 125° of flexion and -25° of extension.

**Figure 9:**
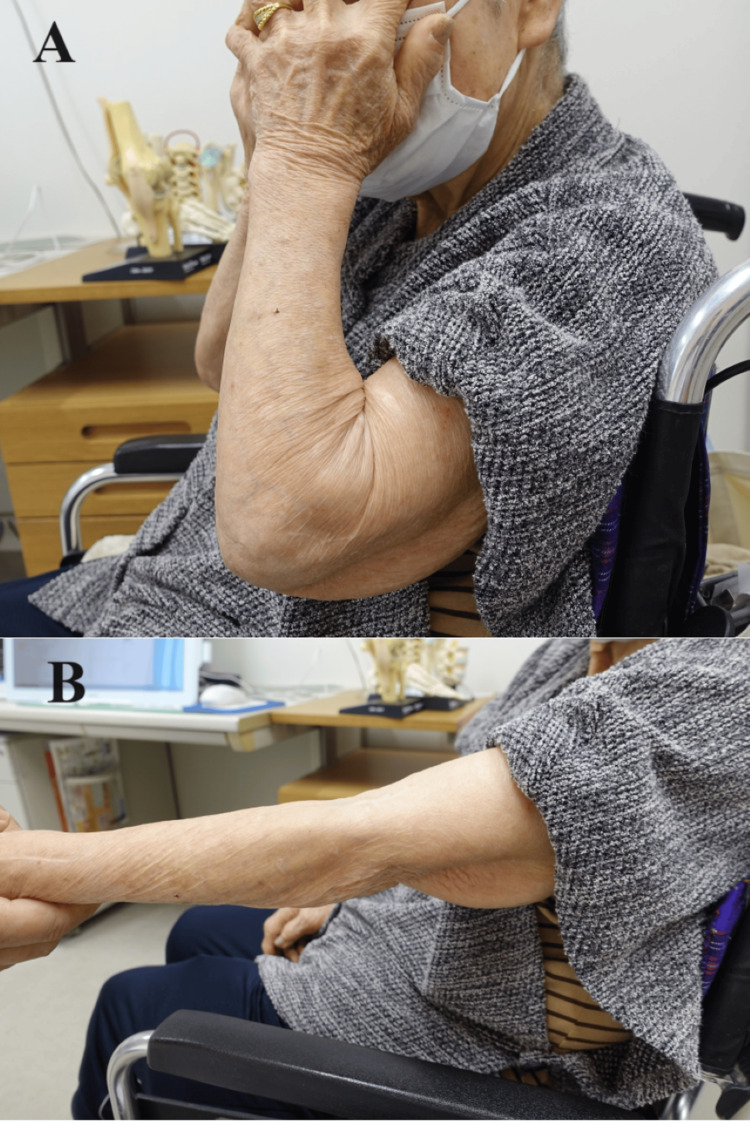
Case two at four years after surgery The patient could (A) wash her face without hindrance and (B) extend her elbow joint.

**Figure 10:**
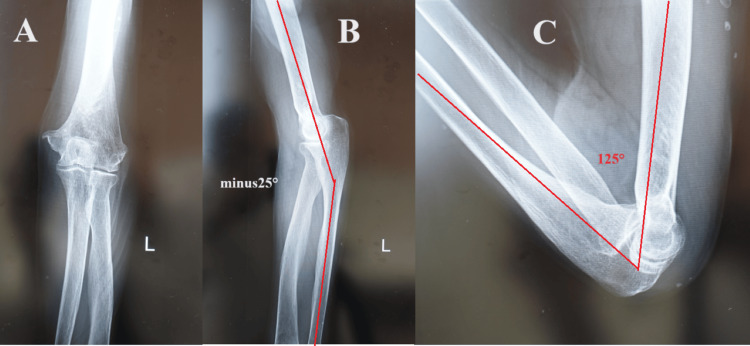
Left elbow X-ray images of case two at four years after surgery (A) Anteroposterior view showing complete bone fusion; (B,C) side views showing the range of motion of the elbow joint was (B) -25° of extension and (C) 125° of flexion.

Four years after surgery, she performed ADL movements, such as washing her face without hindrance (Figure 9), and her ROM was maintained (Figure 10).

## Discussion

Humeral transcondylar fractures are prone to false joints with conservative treatment. According to Noguchi, false joints can be caused by an intracapsular fracture, a small contact area of the fracture, strong instability, and bone fragility due to osteoporosis [1]. As stated by John et al., additional causes include flat fractures with irregularity and small distal bone fragments with minimal soft tissue adhesion [2]. Therefore, surgical treatment is often required in such cases, in which accurate reduction and firm fixation are crucial, as is avoiding injury to the soft tissues. A study by Kusashima et al. reported that ulnar neuropathy was the most important factor for ROM restriction after distal humeral fracture surgery [3]. It was recommended that invasion of the inner condyle and the vicinity of the ulnar nerve should be avoided during the operation. Naka et al. stated that inserting Kirschner wire for percutaneous pinning, which has the advantage of not directly expanding the fracture site, must avoid the olecranon fossa and coronoid process fossa, resulting in poor fixation [4].

In percutaneous pinning, the insertion of the pin avoids the articular cartilage. One pin is struck from the inner surface of the inner condyle, and one pin from the outside of the external condyle in an oblique proximal direction, with the inner pin piercing the bone cortex outside the diaphysis and the outer pin penetrating the bone cortex inside the diaphysis. Therefore, when fixation is emphasized, the medial pin tends to be easily inserted through the ulnar nerve groove, and the insertion of distal bone fragments is very limited when trying to avoid the humeral fossa. Therefore, treatment with percutaneous pinning is likely to fail [5].

Our transarticular cartilage retrograde intramedullary pinning surgery is an excellent surgical method that addresses all the abovementioned problems. As our surgical method does not directly approach the fracture area, it is less invasive to soft tissues. In our method, multiple nails are passed through a narrow medullary cavity so the reduction is accurate, and the fixation force is strong. Furthermore, there is no nail insertion near the cubital tunnel or ulnar nerve, and we do not approach the cubital tunnel area, so there is no chance of damaging the ulnar nerve.

Here is an overview of our surgical methods for humeral transcondylar fracture. Place the upper limbs on top of the trunk in a supine position and put the elbow in a flexed position. Place two short longitudinal incisions on the dorsal side of the elbow joint under axillary nerve block and local anesthesia. I penetrate sharply and reach the distal articular cartilage plane of the humerus. A small hole is made on the surface of the joint, and the nail is driven retrograde using an X-ray fluoroscopy. For the nails, I use a lightly bent tip of Kirschner steel wire. The nail is advanced into the medullary cavity of the distal bone fragment, and the tip from the fracture line is inserted again into the medullary cavity of the proximal bone fragment. Progress as far as possible in the intrathecal cavity. Insert multiple nails from the external and internal condyles.

One of the features of our surgery is two short skin incisions on the dorsal side of the elbow joint in which a small hole is formed in the articular cartilage, and there is almost no invasion of the ulnar nerve or soft tissue. Another feature is the multiple intramedullary nails that pass through the narrow intrathecal cavity of the fracture area. The nails, which occupy a significant portion of the medullary cavity, provide a strong fixation of the fracture and precise reduction of the dislocated bone fragments.

In our transarticular cartilage retrograde intramedullary pinning surgery, we use a cast for six weeks postoperatively. Although the occurrence of elbow joint contracture during cast fixation can be a problem, this method, which is less invasive to soft tissues, is unlikely to cause ROM restriction that interferes with ADL. Muraji recommends five to six weeks of casting at 100°-110° of flexion for conservative treatment of supracondylar fractures [5]. Tomori et al. performed conservative treatment for humeral transcondylar fractures in the elderly with few dislocations; bone fusion was achieved at six to 7.5 weeks, and sufficient joint ROM was obtained [6]. Murata [7] and Nishinaka et al. [8] have stated that if the flexion of the elbow joint is ≥120°, ADL involving the upper body can be performed without any problems. In the current study, all patients had a sufficient flexion ROM of ≥120° postoperatively, and there was no problem with ADL involving the upper body.

## Conclusions

Transarticular cartilage retrograde intramedullary pinning surgery for humeral transcondylar fractures is an excellent surgical method that is less invasive to soft tissues, has accurate reduction and strong fixation, and does not damage the ulnar nerve. As a result, reliable bone fusion and an effective ROM of the elbow joint in daily life can be realized.
